# Determinants of tuberculosis presentation and successful treatment outcome among tuberculosis patients in Geita, Tanzania

**DOI:** 10.1371/journal.pone.0346249

**Published:** 2026-04-07

**Authors:** Jackline Anase Nlula, Sembakutti Samita, Etienne Yuh Jam, Bernard Ngowi, Ellen Mitchell, Anthony Enimil

**Affiliations:** 1 Department of Public Health, Karatu District Hospital, Arushua, Tanzania; 2 Department of Biostatistics, University of Peradeniya, Peradeniya, Central Province, Sri Lanka; 3 Department of Biochemistry, The University of Bamenda, Bamenda, Northwest Region, Cameroon; 4 University of Health and Allied Sciences, Dar es Salaam, Tanzania; 5 Institute of Tropical Medicine, Antwerpen, Belgium; 6 Department of Paediatrics & Infectious Diseases, Kwame Nkrumah University of Science and Technology (KNUST), Kumasi, Ashanti Region, Ghana; Gulu University, UGANDA

## Abstract

The Geita Region of Tanzania is known as critical focal point for tuberculosis (TB) control due to its persistently high disease burden and unique socio-economic landscape. Despite this, limited research has explored how sociodemographic and clinical factors influence TB presentation (pulmonary versus extrapulmonary) and successful treatment outcome. This study aimed to identify these factors within this community to inform targeted interventions. We conducted a retrospective cohort study of TB cases aged 15 years and above, who were notified in Geita Region from January 2017 to December 2020. Data were obtained from the national TB program registers, and included sociodemographic, clinical, and treatment information. The primary outcomes were TB presentation (Pulmonary vs. Extrapulmonary) and treatment outcome, categorized as successful (a composite of “cured” and “treatment completed”) or unsuccessful (death, failure, and loss to follow-up). Multivariable binary logistic regression was applied to assess associations with TB presentation and treatment outcomes, including potential interaction effects between factors. Statistical analyses were performed using SAS OnDemand for initial data cleaning, and Stata 17.0 for all statistical modeling. A total of 13,107 TB cases were included, of which 12,032 (91.9%) pulmonary TB and 1,058 (8.1%) extrapulmonary TB. Logistic regression showed workplace significantly associated with presentation: prisoners had lower odds of pulmonary TB compared to miners (OR 0.26, 95% CI 0.12–0.58). Community-referred cases had higher odds of pulmonary TB than self-referred cases (OR 1.24, 95% CI 1.06–1.45). New TB cases had lower odds of pulmonary TB compared with relapse (OR 0.33, 95% CI 0.15–0.75). Age × sex and HIV × sex interactions associated with differential presentation risks. Treatment success was achieved in 12,385 patients (95.5%), while 581 (4.5%) experienced unsuccessful outcomes. Predictors of successful treatment included age < 60 years (OR 1.31, 95% CI 1.05–1.65), new TB status (OR 2.45, 95% CI 1.53–3.93), and HIV-negative status (OR 1.90, 95% CI 1.53–2.37). Workplace, referral source, and HIV status significantly influence TB presentation and treatment outcomes. Implementation targeted interventions, including strengthened occupational health policies, enhanced community-based screening, and focused strategies for high-risk groups is essential to improving TB control in Tanzania.

## Introduction

Tuberculosis (TB) remains one of the world’s deadliest infectious diseases, despite being curable. In 2024, TB caused approximately 1.23 million deaths globally, highlighting its ongoing threat to public health [1]. The disease burden is particularly severe in Sub-Saharan Africa (SSA), which accounts for over 25% of global TB cases and reports the highest rates of HIV-associated Tanzania is classified among the 30 high-burden TB countries, with an estimated incidence of 183 cases per 100,000 population in 2023 [2]. Many countries, including Tanzania, continue to encounter significant obstacles in TB control. While global initiatives have reduced TB incidence in certain regions, persistent challenges, such as treatment failure and ongoing transmission, persist [3]. These factors impede national progress toward achieving the World Health Organization’s End TB Strategy, which seeks to substantially reduce TB incidence and mortality by 2030 [4].

TB primarily manifests as a pulmonary disease, affecting the lungs. However, if the infection is not contained, it may disseminate to other organs, resulting to, extrapulmonary TB. This form is often more severe and harder to diagnose and treat [5,6]. For instance, when TB infects the brain, it can cause life-threatening complications [7]. The clinical manifestation of TB is determined by a complex interplay of host and environmental factors [8]. Although HIV co-infection is a well-established biological factor that significantly complicates treatment and increases mortality risk [9,10], sociodemographic and structural determinants are increasingly recognized as crucial influences [11,12]. In Tanzania, marked regional disparities in treatment success rates, such as, 99% in Kagera compared to approximately 90% in Geita, underscore the significant impact of local contexts [13]. These disparities suggests underlying causes that extend beyond biology, likely rooted in environmental, occupational, and health system influences [8].

Geita is a region located in north-western Tanzania, is defined by its extensive gold mining industry, which shapes its demographic and health profile [14,15]. The region bears a high TB burden, with an estimated prevalence of 274 per 100,000, exceeding the national average [2,16]. Mining communities are recognized as high-risk environments where factors such as occupational dust exposure, overcrowding in work and living spaces, and a highly mobile workforce converge to create a perfect storm for TB transmission and progression [17]. Furthermore, the presence of distinct sub-communities within this context, such as prison staff and miners, each with unique exposure profiles, adds another layer of complexity to the local TB epidemiology.

Despite the recognition of these challenges, a critical knowledge gap persists. Existing studies in Tanzania have often focused narrowly on specific aspects, such as multidrug-resistant TB or prevalence within single communities [18–20]. There is a striking lack of comprehensive research that simultaneously analyzes how the distinct occupational groups, referral pathways, and clinical characteristics prevalent in a mining region interact to determine both the initial presentation of TB (pulmonary versus extrapulmonary) and the ultimate treatment outcome. Understanding these interconnected determinants is essential to moving beyond a one-size-fits-all approach to TB control

The primary contribution of this study lies in the integrated analysis of sociodemographic, clinical, and health-system factors; along with their interactions on both TB presentation and treatment outcome within the defined, high-burden context of Geita’s community. Therefore, we utilised routine data from Tanzania’s National Tuberculosis and Leprosy Programme (NTLP) to identify the key factors, including occupational group, referral pathway, HIV status, and demographic characteristics that influence TB presentation and treatment success in Geita. By offering a comprehensive perspective, these findings are intended to inform the development of targeted, effective, and context-specific interventions to improve TB control in Geita and other regions with high TB prevalence.

## Materials and methods

### Study design and setting

This retrospective cohort study analyzed routine program data from the Geita Region in northwestern Tanzania, which borders Lake Victoria. The study period extended from January 1, 2017, to December 31, 2020. The Geita Region encompasses approximately 20,054 km² and has a population of about 2 million people [15]. Its economy is primarily based on a large gold mining industry that attracts a substantial migratory workforce [14,15]. This region was chosen due to its high tuberculosis burden, with an estimated prevalence of 274 per 100,000, exceeding the national average and its status as a representative rural area where TB epidemiology is influenced by geographic and socio-economic factors [16]. Notable determinants include significant occupational exposures, prison overcrowding, and public health challenges associated with serving a dispersed and mobile population, particularly in remote areas with limited healthcare access. These characteristics reflect broader national TB trends [16,21].

### Patients’ characteristics

The study population comprised of TB patients aged 15 years and older, who were notified and treated at health facilities in the Geita Region during the study period. The minimum age of 15 years was chosen because occupational status served as a key exposure variable, and individuals younger than 15 years are generally not engaged in formal employment or working formally as miners in Tanzania [22]. TB was defined and confirmed according to the standardized Tanzanian National Tuberculosis and Leprosy Programme (NTLP) guidelines [23], which includes both bacteriological confirmation, such as through smear microscopy or Xpert MTB/RIF assay and clinical diagnosis based on a combination of symptoms, signs, and radiological evidence, followed by a clinician’s decision to initiate a full course of TB treatment. Rifampicin-resistant TB (RR-TB) was identified through routine programmatic diagnostic testing, primarily using the Xpert MTB/RIF assay, in accordance with national guidelines. Cases confirmed as RR-TB were excluded from the analysis of drug-susceptible TB treatment outcomes.

The initial dataset comprised all notified TB cases from Geita Region recorded in the national NTLP database, including patients aged 15 years and older with confirmed tuberculosis diagnosis and documented treatment initiation between January 2017 and December 2020. Exclusion criteria were: (a) patients transferred in from other regions (to ensure complete treatment outcome data from Geita), (b) patients diagnosed with rifampicin-resistant TB (RR-TB) (due to their distinct treatment pathways and outcomes), and (c) records with missing data for primary exposure or outcome variables.

### Sample size estimation and sampling method

This study employed a total population sampling approach, which included all notified tuberculosis cases in the Geita Region from January 2017 to December 2020 that met the eligibility criteria. As this was a complete enumeration of the target population over the four-year period, no formal sample size calculation was required. This approach eliminates sampling bias and provides sufficient statistical power to detect meaningful associations [24]. Post-hoc power calculations are not indicated for census-based studies, as the sample represents the complete target population rather than a subset [25].

### Data collection and research Tool

The de-identified dataset used for this study was provided by the Tanzania National Tuberculosis and Leprosy Programme (NTLP) on January 14, 2023. The NTLP, serving as the data custodian, conducted the initial extraction of the raw, regional-level data and performed de-identification prior to its release for research purposes. The authors were not involved in the initial data extraction or de-identification and did not have access to identifiable patient information at any stage. Upon receipt, the research team undertook a multi-step process to prepare the data for analysis. This process included data cleaning and preparation throughout February 2023, and comprehensive quality assessment that included consistency checks, validation of variable ranges, and the removal of duplicate records. Duplicate records were identified by cross-checking unique patient identifiers and verifying combinations of variables including date of birth, facility registration number, and treatment start date. Where duplicates were confirmed, the entry with complete outcome data was retained. Data management was conducted using Microsoft Excel and SAS OnDemand. After cleaning, the finalized dataset was analyzed using Stata 17.0 (StataCorp, Texas, USA) from March to April 2023. All descriptive statistics and regression modeling were performed during this period. Missing data were minimal, and were addressed through a complete case analysis.

### Definition of study variables

The primary outcomes assessed in this study were tuberculosis (TB) presentation and treatment outcome, both were defined according to standardized international guidelines [26]. TB presentation was measured based on the definitive diagnosis recorded in the patient’s medical record at the time of treatment initiation, following standardized NTLP guidelines [23]. TB presentation was categorized as either pulmonary TB (PTB), referring to disease primarily affecting the lungs, or extrapulmonary TB (EPTB), indicating manifestations outside the lungs such as lymph nodes, pleura, spine, and central nervous system, based on clinical presentation, radiological evidence, and bacteriological confirmation from extrapulmonary sites [23]. Treatment outcome was classified as successful or unsuccessful according to established World Health Organization (WHO) definitions [26]. Successful treatment included patients who were “cured” (with bacteriological confirmation of negativity at the end of treatment) or who “completed treatment” (completed the full course without bacteriological confirmation of cure). Unsuccessful treatment encompassed death, treatment failure, or loss to follow-up [26]. The selection of exposure variables was informed by Andersen’s Behavioral Model to facilitate a comprehensive examination of factors influencing health service utilization [27]. These variables were grouped into three domains: predisposing factors (age, sex, and occupation, specifically miner, healthcare worker, prison staff, or other); enabling factors (referral source, including self-referral, community outreach, HIV clinic [CTC], inpatient department [IPD], or other); and need factors (HIV status, positive or negative, and TB history, new case or relapse). This structured framework supports a multidimensional analysis of the determinants of TB disease manifestation and treatment outcomes.

### Statistical analysis

Data analysis was performed to identify factors associated with two binary outcomes: (a) TB presentation (pulmonary vs. extrapulmonary) and (b) treatment outcome (successful vs. unsuccessful). Descriptive statistics were used to summarise cohort characteristics, reporting frequencies for categorical variables and means with standard deviations for continuous variables.

Multivariable binary logistic regression was used for analysis, as both primary outcomes were dichotomous: TB presentation (pulmonary and extrapulmonary) and treatment outcome(Successful and Unsuccessful). Initial models were specified a priori based on Andersen’s Behavioral Model [27], incorporating predisposing factors (age, sex, occupation), enabling factors (referral source), and need factors (HIV status, TB history). The model for TB presentation also included pre-specified interaction terms (sex × age and sex × HIV) to assess effect modification. Models were fitted using maximum likelihood estimation, and variable significance was assessed using Type III Wald tests (Chi-square) (p < 0.05). Insignificant interaction terms were excluded to produce the final, parsimonious models.

Model adequacy was evaluated using established statistical procedures. Multicollinearity was assessed using Variance Inflation Factors (VIFs), with values less than 5 indicating no concern. Goodness-of-fit was evaluated using the Hosmer-Lemeshow test and deviance statistics. Results are reported as adjusted odds ratios (aOR) with 95% confidence intervals (CI), reflecting the odds of the outcome for each variable category relative to its reference, adjusted for all other covariates. All analyses were performed using Stata 17.0 (StataCorp, Texas, USA).

### Ethical considerations

This study was approved by the National Health Research Ethics Review Committee (NatHREC), Ministry of Health, United Republic of Tanzania (Approval No. NIMR/HQ/R.8a/Vol.IX/4069), dated 03 August 2022. Permission to access routine tuberculosis program data was granted by the Ministry of Health, United Republic of Tanzania (Ref. No PA.87/266/08/06), and the National Tuberculosis and Leprosy Programme. Because the study involved a retrospective review of de-identified data, the ethics committee waived the requirement for informed consent. All data were anonymized before analysis to ensure patient confidentiality.

## Results

### Study population and participant flow

**Fig 1:**
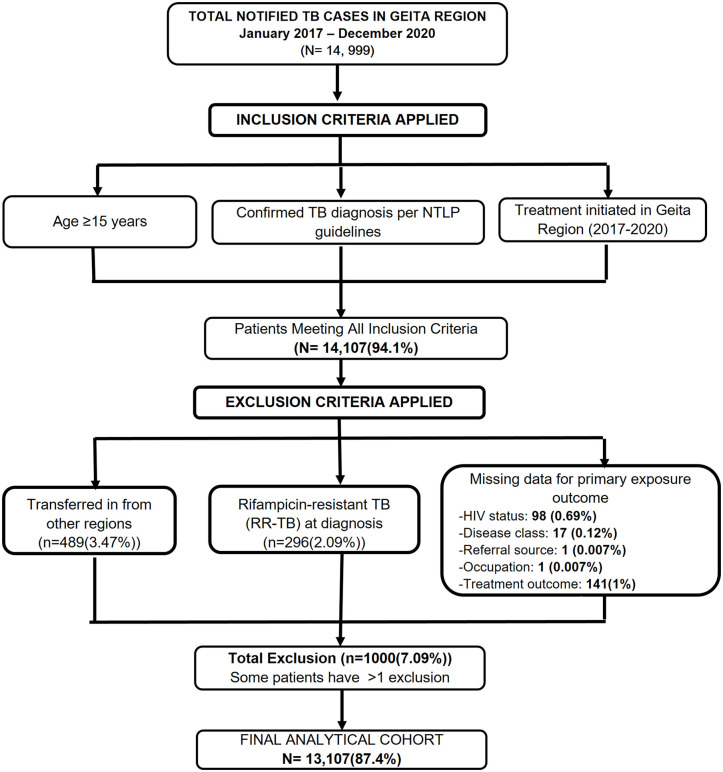
Flowchart of study participant selection and cohort definition for TB cases in the Geita Region, 2017–2020. *This flowchart illustrates the transition from total notified tuberculosis (TB) cases to the final analytical cohort through the sequential application of inclusion and exclusion criteria. It tracks the removal of cases based on age, diagnostic verification, and data completeness.* ***Abbreviations:*** *HIV, Human Immunodeficiency Virus; NTLP, National Tuberculosis and Leprosy Programme; RR-TB, Rifampicin-resistant Tuberculosis; TB, Tuberculosis. Notes: The total exclusions (n = 1,000) represent unique patients, although some individuals met more than one exclusion condition. Missing data accounted for exclusions in HIV status (n = 98), disease classification (n = 17), referral source (n = 1), occupation (n = 1), and treatment outcome (n = 141)*.

A total of 14,999 tuberculosis cases were notified in Geita Region between January 1, 2017 and December 31, 2020. After applying the inclusion criteria (age ≥ 15 years, confirmed TB diagnosis per NTLP guidelines, and treatment initiation in Geita Region), 14,107 patients were eligible for further screening. Of these, patients were excluded for the following reasons: transferred in from other regions (n = 489(3.47%)), rifampicin-resistant TB at diagnosis (n = 296(2.09%), and missing data for primary exposure or outcome variables (HIV status: 98(0.69%); disease classification: 17(0.12%); referral source: 1(0.007%); occupation: 1(0.007%); treatment outcome: 141(1%)). Some patients met more than one exclusion criterion. The final analytical cohort comprised 13,107 patients. The flow of study participant selection is summarised in Fig 1.

### Participant characteristics

The majority of patients were male (63.0%, n = 8,252), and HIV co-infection was identified in 34.2% (n = 4,443) of the cohort. Regarding disease classification, 12,032 (91.9%) cases were pulmonary TB, 1,058 (8.1%) were extrapulmonary TB, and 17 (0.1%) had missing classification data. Most cases (97.9%, n = 12,835) were new diagnoses. The primary referral sources were self-referral (48.4%, n = 6,348) and community referral (28.1%, n = 3,685). Miners constituted 13.0% (n = 1,699) of the occupational cohort. Treatment outcome data were available for 12,966 patients (98.9% of the cohort). Among them, the overall treatment success rate was 95.5% (n = 12,385), while 4.5% (n = 581) had unsuccessful outcomes. The characteristics of the study population are summarized in Table 1.

**Table 1:** Basic characteristics of tuberculosis patients in Geita Region, Tanzania, 2017-2020 (N = 13,107).

Characteristic	Category	Frequency(N)	Percentage (%)
Age group (years)	15-39	4,868	37.1
	40-59	4,859	37.1
	≥60	3,380	25.8
Sex	Male	8,252	63.0
	Female	4,855	37.0
Occupation*	Mining	1,699	13.0
	Healthcare worker	125	1.0
	Prison staff	39	0.3
	Other	11,243	85.8
Referral source*	Self-referral	6,348	48.4
	Community referral	3,685	28.1
	HIV clinic (CTC)	2,526	19.3
	Inpatient dept. (IPD)	186	1.4
	RCHS	7	0.1
	Other	354	2.7
HIV status*	Negative	8,566	65.8
	Positive	4,443	34.1
TB history	New case	12,835	97.9
	Relapse	214	1.6
	Other	58	0.4
Disease classification*	Pulmonary TB	12,032	91.9
	Extrapulmonary TB	1,058	8.1
Treatment outcome*	Successful	12,385	95,5
	Unsuccessful	581	4.5

*Note: Missing data; Occupation (n = 1), Referral source (n = 1), HIV status (n = 98). Percentages are based on available data.* Abbreviations: CTC, Care and Treatment Clinic; RCHS, Reproductive and Child Health Services. Referral sources: Self-referral (patients who presented directly); Community referral (identified by community health workers); HIV clinic (CTC) (referred from HIV care services); Inpatient dept. (IPD) (referred from hospital wards); RCHS (Reproductive and Child Health Services); Other (all other referral pathways)

### Factors associated with tuberculosis presentation

In modelling TB presentation, between the two forms pulmonary and extra-pulmonary, the pulmonary form was modelled. Initially, a logistic regression model incorporating all exposure variables (age group, sex, occupation, referral source, HIV status, and TB history) in an additive structure was fitted. The model provided an adequate fit, with a deviance of 197.95 (P = 0.71) and a Pearson chi-square of 195.76 (P = 0.75). However, Type III analysis revealed that among these exposure variables, only sex, workplace category, referral source, and TB history were significant predictors (P < 0.05). When an extended model including interaction terms was fitted, significant interactions between age × sex (P = 0.0213) and sex × HIV status (P = 0.0045) were identified. Therefore, the final model was fitted using all significant terms from both the initial and extended models. The final model maintained a good fit (deviance = 181.17, P = 0.90; Pearson χ² = 179.74, P = 0.91; df = 207), with all included terms statistically significant. Table 2 displays the adjusted odds ratios (aORs). Notably, Prison staff had lower odds of pulmonary TB compared to miners (aOR = 0.26, 95% CI: 0.12–0.58; P = 0.001), suggesting fundamentally different exposure profiles. Community–referred cases showed higher pulmonary TB odds than self–referred patients (aOR=1.24, 95% CI: 1.06–1.45; P = 0.009), indicating community health workers effectively identify respiratory cases. New TB cases demonstrated 67% lower pulmonary TB odds than relapse cases (aOR=0.33, 95% CI: 0.15–0.75; p = 0.008). The inclusion of interaction effects indicated that these relationships were modified significantly by age and sex, and by sex and HIV status.

**Table 2:** Adjusted odds ratios (aOR) for factors associated with pulmonary vs. extrapulmonary TB presentation.

Factor	Category	aOR	95% CI	p-value
**Occupation (Ref: Miner)**				
	Prison staff	0.26	0.12 - 0.58	**0.001**
	Healthcare worker	0.91	0.46 - 1.80	0.789
	Other	1.05	0.92 - 1.20	0.456
**Referral (Ref: Self-referral)**				
	Community referral	1.24	1.06 - 1.45	**0.009**
	HIV clinic (CTC)	1.09	0.88 - 1.36	0.436
	Inpatient dept. (IPD)	0.68	0.44 - 1.07	0.097
	RCHS	0.59	0.07-4.94	0.626
	Other	0.89	0.65 - 1.22	0.471
**TB history (Ref: Relapse)**				
	New case	0.33	0.15 - 0.75	0.008
	Other history	0.18	0.06 - 0.54	0.002

* Model adjusted for age, sex, HIV status, and interaction terms (sex × age and sex × HIV). Model fit: Deviance P = 0.90, Pearson χ² P = 0.91. Referral sources: Self-referral (patients who presented directly); Community referral (identified by community health workers); HIV clinic (CTC) (referred from HIV care services); Inpatient dept. (IPD) (referred from hospital wards); RCHS (Reproductive and Child Health Services); Other (all other referral pathways)

### Interaction effects of sex with age and HIV status on pulmonary tuberculosis presentation

The significant interaction (P = 0.021) Sex × Age implies that the association between age and TB presentation differ by sex. Among males, odds of having pulmonary TB for those aged 40–59 is 1.26 times that of those who are ≥ 60 years (aOR=1.26, 95% CI: 1.01–1.58), while females showed no significant difference in odds between age groups (Table 3). This likely reflects occupational exposures during peak working years. The Sex × HIV status interaction (P = 0.0045) was of the form that HIV-negative males showed marginally higher pulmonary TB odds than HIV-positive males (aOR=1.21, 95% CI: 0.98–1.49), whereas HIV-negative females had marginally lower odds than their HIV-positive counterparts (aOR=0.80, 95% CI: 0.63–1.03) (Table 3).

**Table 3:** Interaction effects of sex with age and HIV status on pulmonary tuberculosis presentation.

Effect Type	Sex	Stratifying Variable	Category	Adjusted OR*	95% CI
**Age Group**	Female	Age (years)	15-39	1.13	0.87 - 1.46
			40-59	1.04	0.80 - 1.35
			≥60	1.00	Reference
	Male	Age (years)	15-39	0.90	0.72 - 1.12
			40-59	**1.26**	**1.01 - 1.58**
			≥60	1.00	Reference
**HIV Status**	Female	HIV Status	Positive	1.00	Reference
			Negative	**0.80**	**0.63 - 1.03**
	Male	HIV Status	Positive	1.00	Reference
			Negative	**1.21**	**0.98 - 1.49**

*Adjusted for occupation, referral source, and TB history. Global p-value for sex × age interaction = **0.0213**; Global p-value for sex × HIV interaction = **0.0045.**

### Predictors of treatment outcome

In modelling treatment outcome, between the two forms successful and unsuccessful, the successful form was modelled. The fit of the initial logistic regression model with all 5 variables (age group, sex, referral source, workplace, HIV status, and TB history) was satisfactory, with a deviance of 180.58 (P = 0.92) and a Pearson chi–square of 195.10 (P = 0.75) with 209 degrees of freedom. However, significant predictors included age group, referral source, HIV status, and TB history. Extended model with interaction effects detected no significant interaction between factors on treatment outcome. Thus, the final model was fitted with significant factors identified in the initial model. The model’s adequacy remained acceptable in the final fitting, with a deviance of 53.95 (P = 0.59) and a Pearson chi–square of 51.85 (P = 0.67) with 57 degrees of freedom. Table 4 presents the adjusted odds ratios (aORs). Younger patients had significantly better outcomes: Patients aged <40 (aOR=1.30, 95% CI: 1.04–1.62; P = 0.021) and 40–59 (aOR=1.31, 95% CI: 1.05–1.63; P = 0.019) had higher success odds than those with age ≥ 60. HIV–negative status increased success odds compared to HIV–positive (aOR=1.90, 95% CI: 1.53–2.37; P < 0.001). New cases had 2.45-fold higher success than relapses (95% CI: 1.53–3.93). Both Inpatient referrals (aOR = 0.55, 95% CI: 0.31–0.98), and other referral sources (combined non-self/non–IPD) showed lower success odds (aOR = 0.46, 95% CI: 0.30–0.69) compared to that of self-referral (Table 4).

**Table 4:** Odds ratios estimates and confidence intervals for associated factors after fitting the logistic regression model on the proportion of TB treatment outcome.

Factor	Category	aOR	95% CI	p-value
**Age group (Ref: ≥ 60)**				
	15-39 years	1.30	1.04–1.62	**0.021**
	40–59 years	1.31	1.05–1.63	**0.019**
**Referral (Ref: Self-referral)**				
	Inpatient dept. (IPD)	0.55	0.31–0.98	**0.044**
	Other sources*	0.46	0.30–0.69	**<0.001**
**TB history (Ref: Relapse)**				
	New case	2.45	1.53–3.93	**<0.001**
**HIV status (Ref: Positive)**				
	Negative	1.90	1.53–2.37	**<0.001**

*Other sources: Combined non-self/non-IPD referrals. Model fit: Deviance P = 0.59, Pearson χ² P = 0.67.

## Discussion

This study investigated the determinants of tuberculosis (TB) successful treatment outcomes and factors associated with TB presentation in the region of Geita, Tanzania, from 2017 to 2020. Key factors influencing these outcomes include age, occupation, referral pathways, and HIV co-infection. Our findings highlight the complex interplay of individual characteristics and systemic factors affecting TB presentation and treatment success in this high-burden setting. Understanding these determinants can inform targeted interventions to improve TB management in mining communities.

Workplace exposure is a key determinant of tuberculosis (TB) presentation. Prison workers, for instance, exhibit a distinct profile: they have 74% lower odds of developing pulmonary tuberculosis (PTB) but a higher likelihood of extrapulmonary tuberculosis (EPTB) compared to miners (aOR for PTB = 0.26; P = 0.001). This pronounced difference demonstrates how occupation-specific risks influence disease manifestation. Miners are exposed to environments that facilitate respiratory transmission, including silica dust, inadequate ventilation, and crowded underground spaces, which create optimal conditions for pulmonary infections. This exposure likely accounts for their representation in 13% of all PTB cases. These findings are consistent with research from South Africa, where TB rates among miners exceed 7,000 per 100,000, more than ten times the national average [28]. Comparable trends are observed in Zambia and other sub-Saharan African countries, where mining significantly contributes to TB incidence, particularly PTB [29,30]. Although prison settings are recognized as high-risk environments for TB transmission [31] due to overcrowding and limited ventilation, which could theoretically increase PTB risk, the observed lower PTB odds among prison staff compared to miners indicate that additional factors may be involved. These may include differences in health-seeking behavior, access to regular health checks in prison employment, or distinct immunological responses. Further investigation is warranted to elucidate the mechanisms underlying this occupational difference in TB presentation. The substantial proportion of miners among PTB cases reinforces the association between occupational hazards and pulmonary TB. Collectively, these results emphasize the necessity for occupation-specific interventions. In mining communities, strategies such as improved ventilation, health education, and regular screening are essential to reduce PTB transmission. The observed occupational variation in TB presentation confirms that work environments significantly influence both TB risk and disease manifestation, highlighting critical health disparities that require targeted interventions.

Referral pathways significantly influence the presentation of tuberculosis (TB). Community referrals are associated with a 24% higher likelihood of pulmonary TB (PTB) than self-referrals (aOR 1.24; P = 0.009), suggesting that self-referred patients more frequently present with extrapulmonary TB (EPTB). This trend suggests that community health workers (CHWs) are effective at identifying classic respiratory symptoms, such as chronic cough, during active case-finding, thereby facilitating timely PTB diagnosis. In contrast, self-referred cases often involve the non-specific manifestations of EPTB, such as lymphadenopathy or spinal pain, resulting in delayed recognition. These findings align with evidence from Ethiopia, where health extension workers, a type of CHW, significantly increased TB case detection, doubling notifications in supported districts from 64 to 127 per 100,000 population and improving treatment success rates from 76% to 96% over 4.5 years [32]. Similarly, in Mozambique, CHW316 facility-based screening and contact tracing increased TB notifications by 14.6% in intervention areas, while control districts experienced declines [33]. Despite these advances, diagnosing EPTB remains challenging. Reports from Uganda indicate that many EPTB cases self-present after prolonged symptoms [34,35], underscoring the need for targeted community education regarding EPTB signs, such as persistent lymph node swelling.

Age and HIV status are associated with TB presentation, with sex acting as a modifier of these relationships. Males aged 40–59 had 1.26 times higher adjusted odds of PTB than older males (aOR 1.26; 95% CI: 1.01–1.58), suggesting a lower prevalence of EPTB in this group. This pattern likely reflects increased occupational exposures, such as silica dust in mining, during peak working years, consistent with Marçôa et al.’s (2018) findings of higher PTB rates among Portuguese men aged 50–59 [36]. HIV co-infection further complicates these associations: HIV-negative males exhibited higher PTB odds than HIV-positive males, whereas HIV-positive females had higher PTB odds than HIV-negative females. Although estimates within specific groups did not reach statistical significance, the significant sex and HIV interaction (P = 0.0045) demonstrates that biological sex modifies the effect of HIV on TB presentation. A 2018 Ethiopian meta-analysis confirms that HIV-positive individuals have increased TB susceptibility due to immune system weakening [37,38]. These findings suggest that HIV-positive women experience a disproportionate burden of both PTB and EPTB, likely attributable to more advanced immunosuppression. These results underscore the need for occupational lung health programs targeting mining men aged 40–59, enhanced TB screening for HIV-positive women that includes EPTB symptoms such as lymphadenopathy, and clinician training on sex-specific HIV-TB interactions.

The analysis revealed that tuberculosis (TB) history significantly influenced the type of clinical presentation. New TB cases (aOR = 0.33; P = 0.008) demonstrated substantially lower odds of pulmonary TB (PTB) relative to extrapulmonary TB (EPTB) when compared to relapse cases. These results indicate that patients experiencing a TB relapse are significantly more likely to present with pulmonary disease than those with new or undefined prior histories. This finding partially aligns with studies suggesting that relapse may be driven by reactivation of persistent pulmonary foci or undiagnosed drug resistance, which favors PTB [39,40]. In contrast, other research has reported higher EPTB rates in relapse, often associated with immunosuppression or disseminated disease [41]. This discrepancy underscores the complex interplay among local epidemiology, drug resistance patterns, and host factors in relapse presentation. Based on these findings, targeted interventions should prioritize enhanced respiratory symptom screening and sputum testing for all relapse patients, and provide specific education on recognizing pulmonary TB symptoms to encourage prompt care-seeking.

Referrals from hospital inpatient departments (IPD) were associated with a 45% lower likelihood of successful tuberculosis (TB) treatment compared to self-referred patients (aOR 0.55; P = 0.044). This association likely reflects the advanced disease severity, including disseminated extrapulmonary TB (EPTB), HIV co-infection, and malnutrition, which are prevalent among hospitalized individuals and complicate treatment while increasing mortality risk. These findings are consistent with Nidoi et al. (2021) in Uganda, where hospital-referred TB patients experienced higher mortality due to late presentation [42]. Similarly, Mpagama et al. (2020) identified diagnostic delays among hospitalized TB patients with comorbidities in Tanzanian mines [43]. In contrast, these results differ from Ethiopian studies advocating hospital-based screening, underscoring context-specific health system barriers [44]. It is therefore recommended to implement continuous systems to expedite diagnosis and treatment for hospital referrals, conduct regular screening of hospitalized patients for EPTB and comorbidities, and strengthen community-based treatment support following discharge.

HIV-negative patients demonstrated a 90% greater likelihood of successful treatment compared to HIV-positive individuals (aOR 1.90; P < 0.001), highlighting the essential role of immune competence in tuberculosis (TB) cure. HIV-associated immunosuppression impairs treatment response, increases the risk of disseminated extrapulmonary TB (EPTB) such as meningeal or miliary disease, and raises relapse risk due to inadequate drug penetration at sterile sites. These findings are consistent with Gupta-Wright et al. (2022) in Southern Africa, where HIV co-infection was associated with a twofold increase in TB mortality (aHR 2.1), particularly in cases of disseminated EPTB [45]. The increased relapse risk among HIV-positive patients, attributed to persistent subclinical EPTB reservoirs, was further supported by a Ugandan cohort study that reported relapse rates 3.3 times higher than those observed in HIV-negative patients [46].

Patients under 60 years of age demonstrated significantly higher treatment success than those aged 60 years and older. The adjusted odds ratio (aOR) for treatment success among younger patients was 1.30–1.31, representing a 30–31% increase (P < 0.05). Several biological factors may contribute to this disparity, including stronger immune responses that reduce the risk of extrapulmonary tuberculosis (EPTB) dissemination, fewer comorbidities that limit drug interactions, and improved medication tolerance, which decreases the risk of hepatotoxicity—a common cause of treatment interruption in older adults. These findings are consistent with studies from South Korea, where patients under 60 years exhibited higher rates of treatment completion and cure [47]. However, the present results contrast with studies from high-resource settings, such as the Netherlands, which reported smaller age-related effects [48]. This contrast underscores the heightened vulnerability of older adults in resource-limited settings such as Geita.

Newly diagnosed tuberculosis patients demonstrated 2.45 times higher odds of treatment success compared to relapse cases (aOR 2.45; P < 0.001), highlighting retreatment as a critical vulnerability. This outcome is attributed to factors such as undetected drug resistance, extrapulmonary tuberculosis (EPTB) involving sanctuary sites with limited drug penetration, and previous treatment interruptions leading to immunological non-response [49]. These results are consistent with the WHO Global TB Report (2024), which indicates that retreatment cases contribute disproportionately to poor outcomes due to a higher prevalence of drug-resistant TB (16% versus 3.2% in new cases), resulting in lower treatment success for MDR/RR-TB (68% versus 88% for drug-susceptible TB) (2). Recommended interventions include universal drug susceptibility testing, extended EPTB retreatment regimens, decentralized access to second-line drugs, and pharmacist-led adherence support.

This study provides important insights into the determinants of tuberculosis in Geita’s mining communities; however, several limitations should be considered. The retrospective design, which relies on routine program data, limited our ability to include key variables such as socioeconomic status and detailed clinical metrics. The patient cohort was heterogeneous in terms of occupation and referral sources, which, although representative of the region, may have introduced unmeasured confounding. Additionally, the routine national program database aggregated all occupations outside the specified categories of miner, healthcare worker, and prison staff into a single “Other” field, restricting our ability to analyse other occupational groups in detail. While our findings are highly relevant to mining settings in Tanzania, their applicability may differ in regions with distinct health systems or mining operations. Although we did not have complete drug resistance test results for all relapse patients, this limitation does not diminish the significance of our core findings regarding HIV status, referral pathways, age, and occupation-related risks. These factors remain essential for enhancing tuberculosis care in this high-risk population.

### Recommendations

Drawing on our objective to identify determinants of TB presentation and treatment outcomes in Geita, along with our key findings, we propose three targeted interventions. First, mandatory occupational health programs should be implemented at mining sites, prioritizing silica dust control, ventilation improvements, and systematic TB screening for miners, who experience the highest burden of pulmonary TB. Second, community-based case-finding should be strengthened by training health workers to recognize both chronic cough, indicative of pulmonary TB, and subtle symptoms such as lymphadenopathy or persistent pain, which may signal extrapulmonary TB. This approach aims to address the diagnostic gap among self-referred patients. Third, gender-specific TB screening should be integrated into HIV care, with intensified protocols for HIV-positive women, who exhibit a distinct susceptibility to pulmonary TB. Collectively, these interventions address the occupational, community-level, and clinical determinants identified in this study.

Future research should include comprehensive environmental and occupational assessments, such as quantification of silica exposure, and assess the cost-effectiveness of expanding community screening and optimizing referral systems. Additionally, investigation of biological mechanisms underlying sex-specific HIV-TB interactions through biomarker research may provide further insights. Policy recommendations encompass enhanced occupational health interventions, including silica dust control and regular screening; expansion of community health worker-led TB detection; decentralization of molecular diagnostics; and reinforcement of antiretroviral therapy adherence and integrated HIV care. These integrated TB services should prioritize high-risk populations.

## Conclusions

The findings of this study indicate that in the high tuberculosis (TB) burden mining region of Geita, the clinical profile and treatment outcomes of TB are systematically influenced by occupational, clinical, and demographic factors. The elevated risk of pulmonary TB among miners, the demonstrated effectiveness of community referrals in identifying pulmonary cases, and the complex interactions between sex and HIV status highlight the necessity for precisely targeted public health interventions. To reduce the TB burden in this and similar contexts, a shift towards differentiated care strategies is warranted. This approach should include implementing mandatory, periodic occupational health programs in mines, with a focus on silica dust control and active case-finding to protect miners. Additionally, community-based screening should be strengthened by training health workers to identify both classic pulmonary symptoms and subtle extrapulmonary manifestations of TB, thereby minimizing diagnostic delays. Integration of TB-HIV care should also incorporate a gender-specific perspective, ensuring that HIV-positive women receive intensified screening for both pulmonary and extrapulmonary TB. These evidence-based recommendations, directly informed by the study’s results, offer a clear and actionable framework for policymakers and health planners to enhance TB control in Tanzania’s mining communities.

## References

[1] World Health Organization. Global tuberculosis report 2025. 2025. https://www.who.int/teams/global-programme-on-tuberculosis-and-lung-health/tb-reports/global-tuberculosis-report-2025

[2] Global Tuberculosis Report 2024. https://www.who.int/teams/global-programme-on-tuberculosis-and-lung-health/tb-reports/global-tuberculosis-report-2024. Accessed 2025 April 11.

[3] Aldila D, Ramadhan DA, Chukwu CW, Handari BD, Shahzad M, Kamalia PZ. On the role of early case detection and treatment failure in controlling tuberculosis transmission: A mathematical modeling study. Commun Biomath Sci. 2024;7(1):61–86.

[4] Uplekar M, Weil D, Lonnroth K, Jaramillo E, Lienhardt C, Dias HM, et al. WHO’s new end TB strategy. Lancet. 2015;385(9979):1799–801. doi: 10.1016/S0140-6736(15)60570-0 25814376

[5] Baykan AH, Sayiner HS, Aydin E, Koc M, Inan I, Erturk SM. Extrapulmonary tuberculosıs: An old but resurgent problem. Insights Imaging. 2022;13(1):39. doi: 10.1186/s13244-022-01172-0 35254534 PMC8901940

[6] Yu W, Wang Y, Mei J, Hu F, Ji L. Overview of Tuberculosis. Yu W, Lu PX, Tan W. Tuberculosis Control in Migrating Population. Singapore: Springer Singapore. 2020. 1–10.

[7] Malhotra KP, Kulshreshtha D. Pathology of tuberculosis of the nervous system (tuberculous meningitis, tuberculoma, tuberculous abscess). Turgut M, Akhaddar A, Turgut AT, Garg RK. Tuberculosis of the central nervous system. Cham: Springer International Publishing. 2017. 33–53.

[8] Rachwal N, Idris R, Dreyer V, Richter E, Wichelhaus TA, Niemann S. Pathogen and host determinants of extrapulmonary tuberculosis among 1035 patients in Frankfurt am Main, Germany, 2008–2023. Clin Microbiol Infect. 2025;31(3):425–32. 39528087 10.1016/j.cmi.2024.11.009

[9] Letang E, Ellis J, Naidoo K, Casas EC, Sánchez P, Hassan-Moosa R, et al. Tuberculosis-HIV Co-Infection: Progress and challenges after two decades of global antiretroviral treatment roll-out. Arch Bronconeumol. 2020;56(7):446–54. doi: 10.1016/j.arbr.2019.11.013 35373756

[10] Abeid R, Mergenthaler C, Muzuka V, Goodluck A, Nkwabi T, Bigio J, et al. Increasing TB/HIV case notification through an active case-finding approach among rural and mining communities in Northwest Tanzania. J Trop Med. 2022;2022:4716151. doi: 10.1155/2022/4716151 35432549 PMC9007682

[11] Olarewaju SO, Omotola OO, Racheal OB, Olorunnisola AA, Adeyinka A, Adeyemi-Benson O, et al. Factors influencing development of pulmonary tuberculosis among TB patients in Osun State - A case control study. Afr Health Sci. 2025;25(3):12–9. doi: 10.4314/ahs.v25i3.3 41179545 PMC12573656

[12] Scholze AR, Bersi PO, Silva MCD, Martins JT, Melo EC, Galdino MJQ. Prevalence and factors associated with tuberculosis among healthcare workers: a systematic review with meta-analysis. Microbiol Res. 2025;16(8):191.

[13] Njiro BJ, Kisonga R, Joachim C, Sililo GA, Nkiligi E, Ibisomi L, et al. Epidemiology and treatment outcomes of recurrent tuberculosis in Tanzania from 2018 to 2021 using the National TB dataset. PLoS Negl Trop Dis. 2024;18(2):e0011968. doi: 10.1371/journal.pntd.0011968 38359088 PMC10901333

[14] Office of Chief Government Statistician (OCGS), N B of S (NBS). Tanzania Population and Housing Census. Population Distribution by Administrative Areas. Dar es Salaam, Tanzania: Ministry of Finance. 2022. https://www.nbs.go.tz

[15] The United Republic of Tanzania (URT), Ministry of Finance, Tanzania National Bureau of, Statistics and President’s Office - Finance and Planning, Office of the Chief Government, Statistician, Zanzibar. The 2022 Population and Housing Census: Geita Region Basic Demographic and Socio-Economic Profile Report. 2024. https://www.nbs.go.tz/uploads/statistics/documents/en-1738323070-25.%20Geita%20Region%20Socio-Economic%20Profile.pdf

[16] National Tuberculosis and Leprosy Programme, Tazania. TB prevalence in Tanzania. National Tuberculosis and Leprosy Programme Tanzania; 2025 [cited 2025 Jul 13]. https://ntlp.go.tz/tuberculosis/tb-prevalence-in-tanzania/

[17] Chang ST, Chihota VN, Fielding KL, Grant AD, Houben RM, White RG, et al. Small contribution of gold mines to the ongoing tuberculosis epidemic in South Africa: a modeling-based study. BMC Med. 2018;16(1):52. doi: 10.1186/s12916-018-1037-3 29642897 PMC5896106

[18] Mbuya AW, Mboya IB, Semvua HH, Mamuya SH, Msuya SE. Prevalence and factors associated with tuberculosis among the mining communities in Mererani, Tanzania. PLoS One. 2023;18(3):e0280396. doi: 10.1371/journal.pone.0280396 36920939 PMC10016659

[19] Bwire BW, Mbago MCY, Msengwa AS. Spatiotemporal analysis of tuberculosis drug resistance and associated risk factors in Tanzania. Ther Adv Infect Dis. 2025;12:20499361251339576. doi: 10.1177/20499361251339576 40463415 PMC12130660

[20] Zwyer M, Rutaihwa LK, Windels E, Hella J, Menardo F, Sasamalo M. Back-to-Africa introductions of Mycobacterium tuberculosis as the main cause of tuberculosis in Dar es Salaam, Tanzania. Infectious Diseases (except HIV/AIDS). 2022. 10.1371/journal.ppat.1010893 PMC10104295 37014917

[21] The United Republic of Tanzania. Geita Region Socio-Economic Profile. Dar es Salaam, Tanzania: The President’s Office, Regional Administration and Local Government (PO-RALG); 2019. https://www.tanzania.go.tz/egov_uploads/documents/GEITA_REGION_SOCIO_ECONOMIC_PROFILE_2019_sw.pdf

[22] Vice President’s Office. National Action Plan for Artisanal and Small-Scale Gold Mining in the United Republic of Tanzania (2020–2025) [Internet]. Division of Environment; 2020. https://www.mercuryconvention.org/en/resources/national-action-plan-artisanal-and-small-scale-gold-mining-tanzania

[23] National Tuberculosis and Leprosy Programme (NTLP). Manual for Management of Tuberculosis and Leprosy in Tanzania. 7th ed. Dar es Salaam: Ministry of Health, United Republic of Tanzania. 2020.

[24] Banerjee A, Chaudhury S. Statistics without tears: Populations and samples. Ind Psychiatry J. 2010;19(1):60–5. doi: 10.4103/0972-6748.77642 21694795 PMC3105563

[25] Heinsberg LW, Weeks DE. Post hoc power is not informative. Genet Epidemiol. 2022;46(7):390–4. doi: 10.1002/gepi.22464 35642557 PMC9452450

[26] Linh NN, Viney K, Gegia M, Falzon D, Glaziou P, Floyd K, et al. World Health Organization treatment outcome definitions for tuberculosis: 2021 update. Eur Respir J. 2021;58(2):2100804. doi: 10.1183/13993003.00804-2021 34413124

[27] Alkhawaldeh A, ALBashtawy M, Rayan A, Abdalrahim A, Musa A, Eshah N, et al. Application and Use of Andersen’s Behavioral Model as Theoretical Framework: A Systematic Literature Review from 2012–2021. Iran J Public Health. 2023 [cited 2025 Nov 28]: https://publish.kne-publishing.com/index.php/ijph/article/view/13236 10.18502/ijph.v52i7.13236 PMC10430393 37593505

[28] Dharmadhikari A, Smith J, Nardell E, Churchyard G, Keshavjee S. Aspiring to Zero Tuberculosis Deaths among Southern Africa’s Miners: Is There a Way Forward? Int J Health Serv. 2013 Oct;43(4):651–64. 24397232 10.2190/HS.43.4.d

[29] Stuckler D, Basu S, McKee M, Lurie M. Mining and risk of tuberculosis in sub-Saharan Africa. Am J Public Health. 2011;101(3):524–30. doi: 10.2105/AJPH.2009.175646 20516372 PMC3036676

[30] Podewils LJ, Long EF, Fuller TJ, Mwakazanga D, Kapungu K, Tembo M, et al. Zambia Assessment of Tuberculosis (TB) and HIV in the Mines (ZATHIM): Implications for programs and policies. BMC Public Health. 2022;22(1):791. doi: 10.1186/s12889-022-13053-8 35439984 PMC9018205

[31] Nyasulu PS, Hui DS, Mwaba P, Tamuzi JL, Sakala DY, Ntoumi F, et al. Global perspectives on tuberculosis in prisons and incarceration centers - Risk factors, priority needs, challenges for control and the way forward. IJID Reg. 2025;14(Suppl 2):100621. doi: 10.1016/j.ijregi.2025.100621 40201555 PMC11973647

[32] Datiko DG, Yassin MA, Theobald SJ, Blok L, Suvanand S, Creswell J, et al. Health extension workers improve tuberculosis case finding and treatment outcome in Ethiopia: A large-scale implementation study. BMJ Glob Health. 2017;2(4):e000390. doi: 10.1136/bmjgh-2017-000390 29209537 PMC5704104

[33] José B, Manhiça I, Jones J, Mutaquiha C, Zindoga P, Eduardo I, et al. Using community health workers for facility and community based TB case finding: An evaluation in central Mozambique. PLoS One. 2020;15(7):e0236262. doi: 10.1371/journal.pone.0236262 32702073 PMC7377411

[34] Jain R, Gupta G, Mitra DK, Guleria R. Diagnosis of extra pulmonary tuberculosis: An update on novel diagnostic approaches. Respir Med. 2024;225:107601. doi: 10.1016/j.rmed.2024.107601 38513873

[35] Kirenga BJ, Ssengooba W, Muwonge C, Nakiyingi L, Kyaligonza S, Kasozi S, et al. Tuberculosis risk factors among tuberculosis patients in Kampala, Uganda: implications for tuberculosis control. BMC Public Health. 2015;15:13. doi: 10.1186/s12889-015-1376-3 25604986 PMC4311451

[36] Marçôa R, Ribeiro AI, Zão I, Duarte R. Tuberculosis and gender - Factors influencing the risk of tuberculosis among men and women by age group. Pulmonology. 2018;24(3):199–202. doi: 10.1016/j.pulmoe.2018.03.004 29754721

[37] Humayun M, Chirenda J, Ye W, Mukeredzi I, Mujuru HA, Yang Z. Effect of Gender on Clinical Presentation of Tuberculosis (TB) and Age-Specific Risk of TB, and TB-Human Immunodeficiency Virus Coinfection. Open Forum Infect Dis. 2022;9(10):ofac512. doi: 10.1093/ofid/ofac512 36324321 PMC9620549

[38] Navasardyan I, Miwalian R, Petrosyan A, Yeganyan S, Venketaraman V. HIV–TB coinfection: Current therapeutic approaches and drug interactions. Viruses. 2024;16(3):321. 38543687 10.3390/v16030321 PMC10974211

[39] Cardona P-J. Reactivation or reinfection in adult tuberculosis: Is that the question?. Int J Mycobacteriol. 2016;5(4):400–7. doi: 10.1016/j.ijmyco.2016.09.017 27931680

[40] Velayutham B, Chadha VK, Singla N, Narang P, Gangadhar Rao V, Nair S, et al. Recurrence of tuberculosis among newly diagnosed sputum positive pulmonary tuberculosis patients treated under the Revised National Tuberculosis Control Programme, India: A multi-centric prospective study. PLoS One. 2018;13(7):e0200150. doi: 10.1371/journal.pone.0200150 29979738 PMC6034867

[41] Lee H, Kim J. A study on the relapse rate of tuberculosis and related factors in Korea using nationwide tuberculosis notification data. Osong Public Health Res Perspect. 2014;5(Suppl):S8–17. doi: 10.1016/j.phrp.2014.11.001 25861581 PMC4301639

[42] Becker GL, Amuge P, Ssebunya R, Motevalli M, Adaku A, Juma M. Predictors of mortality in Ugandan children with TB, 2016–2021. Int J Tuberc Lung Dis. 2023;27(9):668–74. 37608479 10.5588/ijtld.22.0622 PMC10443779

[43] Mpagama SG, Ezekiel MJ, Mbelele PM, Chongolo AM, Kibiki GS, de Guex KP, et al. Gridlock from diagnosis to treatment of multidrug resistant tuberculosis (MDR-TB) in Tanzania: patients’ perspectives from a focus group discussion. BMC Public Health. 2020;20(1):1667. doi: 10.1186/s12889-020-09774-3 33160327 PMC7648291

[44] Jerene D, Hiruy N, Jemal I, Gebrekiros W, Anteneh T, Habte D. The yield and feasibility of integrated screening for TB, diabetes and HIV in four public hospitals in Ethiopia. Int Health. 2017;9(2):100–4. 28338880 10.1093/inthealth/ihx002

[45] Gupta-Wright A, Fielding K, Wilson D, Van Oosterhout JJ, Grint D, Mwandumba HC, et al. Tuberculosis in hospitalized patients with human immunodeficiency virus: clinical characteristics, mortality, and implications from the rapid urine-based screening for tuberculosis to reduce AIDS related mortality in hospitalized patients in Africa. Clinical Infectious Diseases. 2020;71(10):2618–26. 31781758 10.1093/cid/ciz1133 PMC7744971

[46] Luzze H, Johnson DF, Dickman K, Mayanja-Kizza H, Okwera A, Eisenach K, et al. Relapse more common than reinfection in recurrent tuberculosis 1–2 years post treatment in urban Uganda. int j tuberc lung dis. 2013;17(3):361–7. doi: 10.5588/ijtld.11.0692 23407224 PMC6623981

[47] Lee J, Lim JK, Kim EJ, Lee DH, Kim YK, Yoo SS, et al. Comparison of clinical manifestations and treatment outcome according to age groups in adult patients with miliary tuberculosis. J Thorac Dis. 2018;10(5):2881–9. doi: 10.21037/jtd.2018.04.139 29997953 PMC6006096

[48] Borgdorff MW, Van Der Werf MJ, De Haas PEW, Kremer K, Van Soolingen D. Tuberculosis elimination in the Netherlands. Emerg Infect Dis. 2005;11(4):597–602. 15829200 10.3201/eid1104.041103 PMC3320334

[49] Sevim T, Ataç G, Güngör G, Törün I, Aksoy E, et al. Treatment outcome of relapse and defaulter pulmonary tuberculosis patients. Int J Tuberc Lung Dis. 2002;6(4):320–5. 11936741

